# A Positive Correlation of Basal Metabolic Rate With Significant Liver Fibrosis in Adults With MASLD and Obesity: Results From NHANES 2017–2020

**DOI:** 10.1155/cjgh/3293133

**Published:** 2026-05-09

**Authors:** Shu-qin Zheng, Jing-tao Qin, Yi-shan He, Xue-ming Zhang, Yuan Xue

**Affiliations:** ^1^ Department of Infectious Diseases, Changzhou Third People’s Hospital, Changzhou, China, jsczsy.cn; ^2^ Department of Liver Diseases, Changzhou Clinical College, Xuzhou Medical University, Changzhou, China, xzmc.edu.cn

**Keywords:** basal metabolic rate, body mass index, controlled attenuation parameter, liver fibrosis, metabolic dysfunction–associated steatotic liver disease

## Abstract

**Background and Aim:**

Since the stage of liver fibrosis is closely associated with mortality in nonalcoholic fatty liver disease (NAFLD) and metabolic dysfunction–associated steatotic liver disease (MASLD), it is essential to identify patients at risk of fibrosis progression. Basal metabolic rate (BMR) has gained attention in weight management for individuals with obesity. This study aimed to assess the accuracy of BMR in predicting the severity of fibrosis in patients with NAFLD or MASLD.

**Methods:**

Data from the National Health and Nutrition Examination Survey (NHANES) 2017–2020.03 were analyzed. Noninvasive diagnosis of liver steatosis was performed using the controlled attenuation parameter (CAP) measured by FibroScan. BMR calculated using the Harris–Benedict equation (BMRh) and the Mifflin–St Jeor equation (BMRm) was included in the analysis.

**Results:**

A total of 4184 individuals with MASLD were included, of whom 639 (15.27%) had significant fibrosis. Among 1577 individuals with NAFLD, 223 (14.14%) had significant fibrosis. Participants with significant fibrosis were older and had higher CAP, BMRh, and BMRm values (all *p* < 0.05). Multivariate analysis identified BMR as an independent risk factor for significant fibrosis. In obesity participants with MASLD or NAFLD, both BMRh and BMRm were positively correlated with BMI, CAP, and liver stiffness measurement (all *p* < 0.01). For obesity participants with MASLD or NAFLD, both BMRh‐ or BMRm‐incorporating models had higher AUROCs than aspartate transaminase‐to‐platelet ratio index, fibrosis‐4, and gamma‐glutamyl transpeptidase to platelet ratio in predicting significant fibrosis (all *p* < 0.001).

**Conclusion:**

BMR is elevated in individuals with NAFLD or MASLD and significant liver fibrosis. The novel model combining age, male gender, BMR, aspartate transaminase, white blood cell counts, platelet counts, and diabetes outperformed conventional noninvasive scoring systems in predicting significant fibrosis in individuals with MASLD and obesity.

## 1. Introduction

Nonalcoholic fatty liver disease (NAFLD), with an estimated global prevalence of 32%, is the most common cause of chronic liver disease [1]. Its incidence continues to rise and may progress to liver cirrhosis or hepatocellular carcinoma, posing a significant public health burden. Increasing evidence indicates a strong association between fibrosis stage and NAFLD‐related mortality, making it critical to identify patients at risk of fibrosis progression [2, 3]. A new nomenclature for metabolic dysfunction–associated steatotic liver disease (MASLD), encompassing liver steatosis and metabolic dysfunction–associated steatohepatitis (MASH), has been now widely accepted [4].

In patients with NAFLD, noninvasive scoring systems (NSSs) such as the aminotransferase‐to‐platelet ratio index (APRI), gamma‐glutamyl transpeptidase to platelet ratio (GPR), and fibrosis‐4 (FIB‐4) have limited predictive accuracy [5, 6]. For individuals with MASLD, FIB‐4, and APRI showed better diagnostic values than the hepamet fibrosis score and the NAFLD score [6]. While liver biopsy remains the reference standard, it is invasive and unsuitable for routine monitoring, necessitating more effective noninvasive approaches.

Energy expenditure (EE) plays a vital role in energy balance and weight regulation. EE is influenced by multiple factors, including basal metabolic rate (BMR), age, sex, physical activity, health status, and environmental conditions. BMR, which reflects the energy required to maintain basic physiological functions at rest, constitutes the largest component of daily EE in sedentary individuals. BMR is influenced by body composition, particularly fat mass (FM) and fat‐free mass (FFM), as different tissues and organs exhibit varying energy demands. Prior studies have demonstrated that EE declines with age and varies by sex, potentially due to altered energy metabolism. Age‐related reductions in BMR have been observed in children, adolescents, and older adults, primarily due to reductions in FFM [7, 8]. BMR has thus become a key focus in obesity‐related weight management.

To date, the relationship between EE, BMR, NAFLD, and MASLD progression remains unclear in individuals with obesity. Body composition alone does not fully explain BMR variability in the general population, especially in individuals with severe obesity. Moreover, those with severe obesity are at a higher risk of developing advanced liver fibrosis compared with individuals who are overweight or have normal weight [9]. While in a nonobese Chinese cohort, unanticipated results showed that elevated BMR was an independent risk factor for MASLD incidence [10]. Therefore, it is necessary to investigate the association between BMR, NAFLD, and MASLD progression across different weight categories.

In this study, we assessed the predictive value of BMR for fibrosis severity in patients with NAFLD or MASLD and developed a noninvasive model to evaluate liver fibrosis.

## 2. Materials and Methods

### 2.1. Data Sources

Data on demographic characteristics, physical examinations, laboratory tests, and questionnaires were obtained from the National Health and Nutrition Examination Survey (NHANES) 2017–2020.03 and analyzed. The controlled attenuation parameter (CAP) and liver stiffness measurement (LSM) were assessed using FibroScan model 502 V2 Touch. Individuals who were pregnant, tested positive for viral hepatitis markers, reported heavy alcohol use [11, 12], had insufficient data, or had CAP < 248 dB/m were excluded, as previously described [13]. A total of 1577 individuals with CAP ≥ 248 dB/m were identified as having NAFLD [14, 15]. MASLD was diagnosed in participants with hepatic steatosis and at least one cardiometabolic risk factor, including [2] (1) body mass index (BMI) ≥ 25 kg/m^2^ or waist circumference (WC) ≥ 94 cm for male and ≥ 80 cm for female, (2) fasting plasma glucose ≥ 100 mg/dL or glycated hemoglobin A1c ≥ 5.7% or Type 2 diabetes or drug treatment, (3) blood pressure ≥ 130/85 mmHg or drug treatment, (4) plasma triglycerides (TG) ≥ 150 mg/dL or drug treatment, and (5) high‐density lipoprotein (HDL) ≤ 40 mg/dL for men and ≤ 50 mg/dL for women or drug treatment. Moreover, individuals with secondary causes of steatosis, including excessive alcohol consumption (≥ 210 g/week for men and ≥ 140 g/week for women) and other chronic liver diseases were excluded [2]. After screening, 4184 individuals with MASLD were included in the analysis.

Significant fibrosis was defined as LSM ≥ 8.1 kPa [16]. Participants were stratified into three groups based on BMI: normal weight (BMI < 25 kg/m^2^), overweight (BMI 25–29.9 kg/m^2^), and obesity (BMI ≥ 30 kg/m^2^).

In addition to demographic data and comorbidities, covariates such as alanine aminotransferase (ALT), aspartate aminotransferase (AST), C‐reactive protein (CRP), white blood cell (WBC) count, and platelet count were collected.

### 2.2. Definitions of BMR and NSS

BMI was calculated using the following formula: BMI = weight (kg)/height^2^ (m^2^).

The basal metabolic rate calculated using the Harris–Benedict equation (BMRh) was calculated using the Harris–Benedict equation: BMRh (for men) = 88.362 + 13.397 ∗ weight (kg) + 4.799 ∗ Height (cm)–5.677 ∗ age and BMRh (for women) = 447.593 + 9.247 ∗ weight (Kg) + 3.098 ∗ Height (cm)–4.330 ∗ age; The Basal metabolic rate calculated using the Mifflin–St Jeor equation (BMRm) was calculated using the Mifflin–St Jeor equation: BMRm (for men) = 9.99 ∗ weight (Kg) + 6.25 ∗ Height (cm)–4.92 ∗ age + 5 and BMRm (for women) = 9.99 ∗ weight (Kg) + 6.25 ∗ Height (cm)–4.92 ∗ age–161 [17, 18].

NSSs, including FIB‐4, GPR, and APRI, were calculated as previously described [19].

### 2.3. Statistical Analysis

Data were analyzed using EmpowerStats (https://www.empowerstats.com) and SPSS Version 25.0 (NY, USA). Continuous variables were expressed as medians with interquartile ranges and compared using the Mann–Whitney U test. Categorical variables were presented as frequencies and compared using the chi‐square test. Independent risk factors for significant fibrosis were assessed using multivariate logistic regression analysis. Correlation heatmaps were generated using an online tool (https://hiplot.com.cn). NSSs were compared using the area under the receiver operating characteristic curve (AUROC), analyzed with MedCalc Version 20.1.0 (MedCalc Software, Mariakerke, Belgium). A two‐sided *p* value < 0.05 was considered statistically significant.

## 3. Results

### 3.1. Characteristics of Participants

Among 4184 individuals with MASLD, 639 had significant fibrosis and 3545 had nonsignificant fibrosis (Table 1). More participants in the significant fibrosis group were male (*χ* ^2^ = 10.333 and *p* = 0.001). Participants with significant fibrosis were older and had higher rates of diabetes (all *p* < 0.01). Levels of CAP, LSM, GPR, APRI, FIB‐4, BMI, BMRh, BMRm, ALT, AST, CRP, and WBC were significantly higher (all *p* < 0.01), while the platelet counts were significantly lower in participants with significant fibrosis (*p* < 0.01).

**TABLE 1:** Characteristics of participants with MASLD and significant fibrosis.

	**Non-significant fibrosis (*n* = 3545)**	**Significant fibrosis (*n* = 639)**	***p*** **value**

Male, *n*(%)	1808 (51.001%)	370 (57.903%)	0.001
Age, yrs	53.000 (39.000–64.000)	57.000 (45.000–66.000)	< 0.001
Hypertension, *n* (%)	956 (26.968%)	160 (25.039%)	0.310
Diabetes, *n* (%)	340 (9.591%)	139 (21.753%)	< 0.001
BMI (kg/m^2)^	30.800 (27.400–35.100)	36.900 (31.700–42.800)	< 0.001
BMRh	1649.884 (1441.757–1900.851)	1858.841 (1591.283–2180.675)	< 0.001
BMRm	1593.145 (1373.582–1806.088)	1774.005 (1528.849–2028.166)	< 0.001
CAP (dB/m)	296.000 (272.000–329.000)	335.000 (299.000–369.000)	< 0.001
GPR	9.620 (6.490–14.890)	13.020 (8.30–25.110)	< 0.001
APRI	0.216 (0.163–0.291)	0.252 (0.180–0.377)	< 0.001
FIB‐4	0.910 (0.597–1.307)	1.081 (0.698–1.577)	< 0.001
Stiffness (kPa)	5.100 (4.200–6.050)	10.600 (8.900–14.600)	< 0.001
ALT (U/L)	19.000 (14.000–28.000)	23.000 (16.000–37.000)	< 0.001
AST (U/L)	19.000 (16.000–24.000)	21.000 (17.000–29.000)	< 0.001
CRP (mg/L)	2.410 (1.100–4.955)	3.700 (1.750–7.330)	< 0.001
WBC, x 10^9^/L	7.100 (5.900–8.600)	7.500 (6.300–9.100)	< 0.001
Platelet, x 10^9^/L	246.000 (208.000–289.000)	232.000 (193.000–278.000)	< 0.001

*Note:* Data were expressed as median (IQR) for continuous variables and *n* (%) for categorical values and were compared using the Mann–Whitney *U* test or Chi‐square test. MASLD, metabolic dysfunction–associated steatotic liver disease; BMRh, basal metabolic rate calculated using the Harris–Benedict equation; BMRm, basal metabolic rate calculated using the Mifflin–St Jeor equation; ALT, alanine aminotransferase; AST, aspartate transaminase; APRI, aminotransferase‐to‐platelet ratio index; FIB‐4, fibrosis‐4; GPR, gamma‐glutamyl transpeptidase to platelet ratio.

Abbreviations: BMI, body mass index; CAP, controlled attenuated parameter; CRP, C‐reaction protein; WBC, white blood cell.

Data from 1577 individuals with NAFLD were analyzed, including 223 with significant fibrosis and 1354 with nonsignificant fibrosis (Table 2). Participants with significant fibrosis were older and had higher rates of hypertension and diabetes (all *p* < 0.05). Compared with those with significant fibrosis, participants without significant fibrosis had lower levels of CAP, LSM, GPR, APRI, FIB‐4, BMI, BMRh, BMRm, ALT, AST, CRP, TC, TG, and WBC and higher levels of platelet count (all *p* < 0.01).

**TABLE 2:** Characteristics of participants with NAFLD and significant fibrosis.

	**Nonsignificant fibrosis (*n* = 1354)**	**Significant fibrosis (*n* = 223)**	***p*** **value**

Male, *n*(%)	671 (49.557%)	118 (52.915%)	0.353
Age, yrs	53.000 (37.000–64.000)	57.000 (43.000–66.500)	0.003
Hypertension, *n* (%)	308 (22.747%)	65 (29.148%)	0.037
Diabetes, *n* (%)	221 (16.3%)	90 (40.4%)	< 0.001
BMI (kg/m^2^)	30.800 (27.300–35.275)	37.400 (32.500–44.350)	< 0.001
BMRh	1660.794 (1443.821–1897.662)	1892.275 (1624.135–2193.112)	< 0.001
BMRm	1604.188 (1376.781–1808.062)	1808.500 (1556.188–2068.312)	< 0.001
CAP (dB/m)	294.000 (271.000–329.000)	342.000 (308.500–378.000)	< 0.001
GPR	9.410 (6.540–14.335)	13.360 (8.460–23.530)	< 0.001
APRI	0.200 (0.150–0.268)	0.240 (0.170–0.350)	< 0.001
FIB‐4	0.900 (0.570–1.260)	1.060 (0.655–1.505)	< 0.001
Stiffness(kPa)	5.000 (4.200–5.900)	10.800 (9.000–15.150)	< 0.001
ALT (U/L)	19.000 (14.000–27.000)	24.000 (16.000–36.000)	< 0.001
AST (U/L)	19.000 (15.000–23.000)	21.000 (17.000–28.000)	< 0.001
CRP (mg/L)	2.465 (1.103–4.968)	3.910 (1.945–8.530)	0.001
WBC, x 10^9^/L	6.700 (5.500–8.100)	7.400 (6.000–8.550)	< 0.001
Platelet, x 10^9^/L	245.000 (206.000–287.750)	231.000 (189.000–280.000)	0.008

*Note:* Data were expressed as median (IQR) for continuous variables and *n* (%) for categorical values and were compared using Mann–Whitney U test or Chi‐square test. BMRh, basal metabolic rate calculated using the Harris–Benedict equation; BMRm, basal metabolic rate calculated using the Mifflin–St Jeor equation; ALT, alanine aminotransferase; AST, aspartate transaminase; FIB‐4, fibrosis‐4; GPR, gamma‐glutamyl transpeptidase to platelet ratio.

Abbreviations: APRI, aminotransferase‐to‐platelet ratio index; BMI, body mass index; CAP, controlled attenuated parameter; CRP, C‐reaction protein; NAFLD, nonalcoholic fatty liver disease; WBC, white blood cell.

### 3.2. Association Between BMR and Liver Stiffness

In participants with MASLD, BMRh and BMRm exhibited significant negative correlations with age (*r* = −0.404 and −0.428, respectively; both *p* < 0.01), whereas positive associations were found with BMI, LSM, CAP, ALT, AST, CRP, and WBC (all *p* < 0.01). LSM was also positively correlated with BMI, CAP, ALT, AST, CRP, and WBC (all *p* < 0.01) and negatively correlated with platelet counts (all *p* < 0.01) (Figure 1(a)).

FIGURE 1Correlation of BMR with clinical parameters (a) and comparison between Model_BMRh, Model_BMRm, and noninvasive scoring systems in individuals with MASLD (b). ALT, alanine aminotransferase; AST, aspartate transaminase; BMI, body mass index; CAP, controlled attenuated parameter; CRP, C‐reactive protein; BMRh, basal metabolic rate calculated using the Harris–Benedict equation; BMRm, basal metabolic rate calculated using the Mifflin–St Jeor equation; MASLD, metabolic dysfunction–associated steatotic liver disease; APRI, AST‐to‐PLT ratio index; FIB‐4, fibrosis‐4; GPR, gamma‐glutamyl transpeptidase to platelet ratio; WBC, white blood cell.

**Figure:**
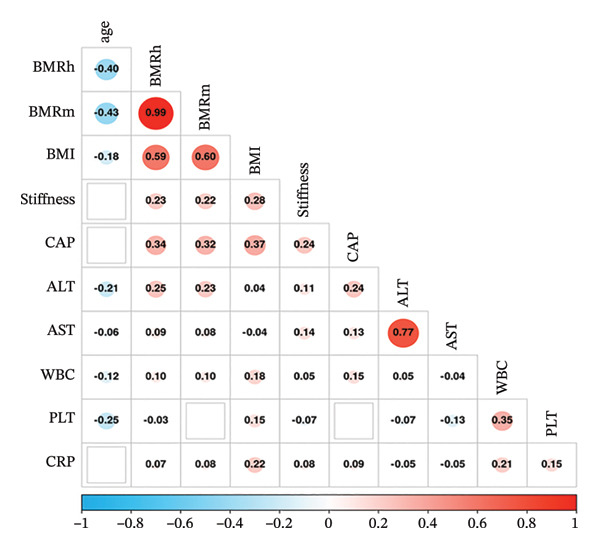
(a)

**Figure:**
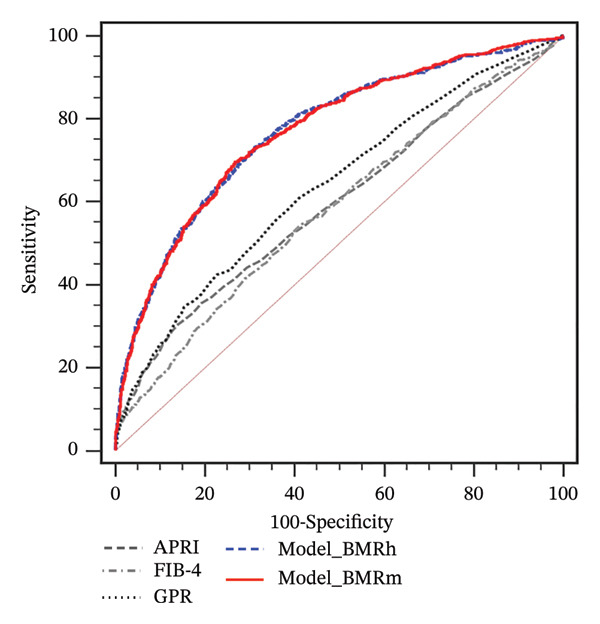
(b)

Then, the participants with MASLD were divided into three subgroups, including obesity (*n* = 2496), overweight (*n* = 1301), and normal weight (*n* = 387). In the three subgroups, both BMRh and BMRm were positively correlated with BMI and negatively correlated with age (both *p* < 0.01). In individuals with MASLD and obesity, both BMRh and BMRm were positively correlated with LSM and CAP (both *p* < 0.01). In individuals with MASLD and overweight, neither BMRh nor BMRm showed a significant correlation with LSM or CAP (all *p* > 0.05). In normal‐weight participants with MASLD, both BMRh and BMRm showed positive correlations with LSM (*p* = 0.031 and 0.019, respectively) but demonstrated no significant relationship with CAP (*p* = 0.942 and 0.935).

Similar results were found in participants with NAFLD, both BMRh and BMRm were negatively correlated with age (*r* = −0.407 and −0.439, respectively; both *p* < 0.01) and positively correlated with BMI, LSM, CAP, ALT, AST, CRP, and WBC (all *p* < 0.01) (Figure 2(a)).

FIGURE 2Correlation of BMR with clinical parameters (a) and comparison between Model_BMRh_NAFLD, Model_BMRm_NAFLD, and noninvasive scoring systems in individuals with NAFLD (b). ALT, alanine aminotransferase; AST, aspartate transaminase; BMI, body mass index; CAP, controlled attenuated parameter; CRP, C‐reactive protein; BMRh, basal metabolic rate calculated using the Harris–Benedict equation; BMRm, basal metabolic rate calculated using the Mifflin–St Jeor equation; NAFLD, nonalcoholic fatty liver disease; APRI, AST‐to‐PLT ratio index; FIB‐4, fibrosis‐4; GPR, gamma‐glutamyl transpeptidase to platelet ratio; WBC, white blood cell.

**Figure:**
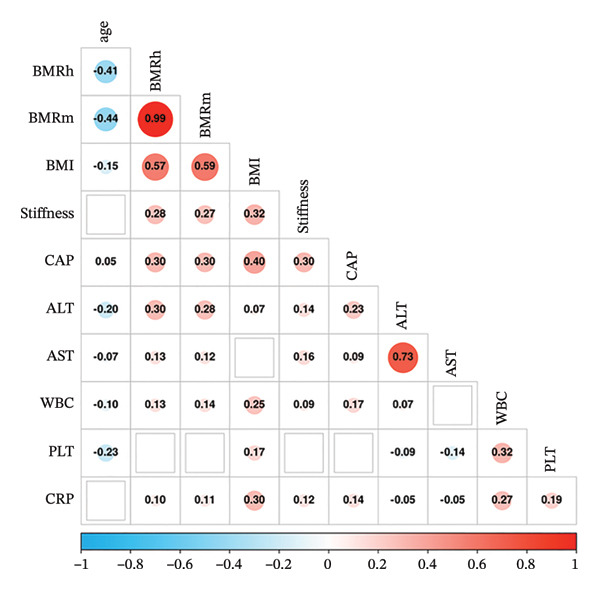
(a)

**Figure:**
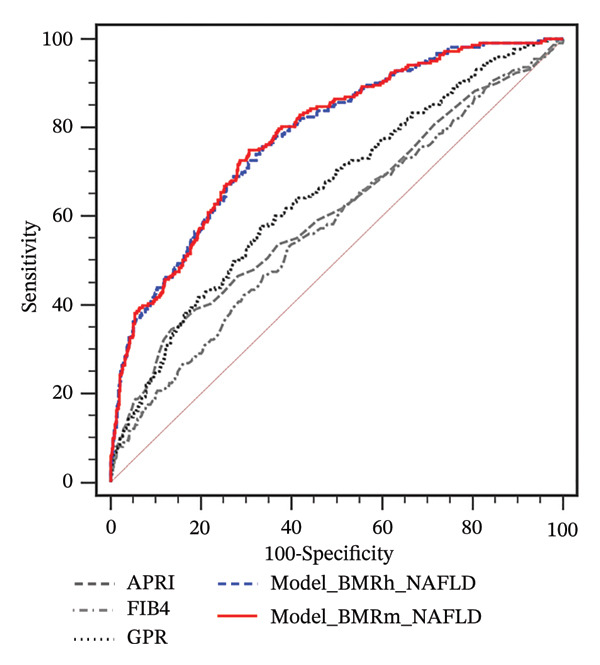
(b)

Then, participants with NAFLD were divided into three subgroups: obesity (*n* = 949), overweight (*n* = 456), and normal weight (*n* = 172). In the obesity group, both BMRh and BMRm were positively correlated with BMI, ALT, AST, CAP, and LSM (all *p* < 0.05). However, in the overweight and normal weight groups, neither BMRh nor BMRm showed significant correlation with CAP or LSM (all *p* > 0.05).

### 3.3. Development of Two New Models for Predicting Significant Fibrosis in MASLD

Univariate analysis showed that age, male, BMRh, ALT, AST, CRP, WBC, platelet count, and diabetes were associated with significant fibrosis (all *p* < 0.01). Multivariate analysis confirmed that age, male, BMRh, AST, WBC, platelet count, and diabetes (all *p* < 0.01) were independent risk factors for significant fibrosis. When BMRm was substituted for BMRh, multivariate analysis showed that age, male, BMRm, AST, WBC, platelet count, and diabetes were independent predictors of significant fibrosis (all *p* < 0.01) (Table 3).

**TABLE 3:** Risk factors for significant fibrosis in participants with MASLD.

Variables	Univariate			Multivariate		
	Odds ratio	95% CI	*p*	Odds ratio	95% CI	*p*
Age	1.012	1.007–1.017	< 0.001	1.043	1.036–1.051	< 0.001
Male	1.321	1.115–1.567	0.001	0.287	0.224–0.369	< 0.001
BMRh	1.002	1.001–1.002	< 0.001	1.003	1.003–1.003	< 0.001
ALT	1.019	1.014–1.023	< 0.001			
AST	1.031	1.024–1.038	< 0.001	1.037	1.030–1.045	< 0.001
CRP	1.016	1.008–1.025	< 0.001			
WBC	1.085	1.045–1.126	< 0.001	1.092	1.045–1.141	< 0.001
Platelet	0.997	0.996–0.998	< 0.001	0.997	0.996–0.999	0.002
Diabetes (1,0)	0.382	0.307–0.475	< 0.001	0.445	0.348–0.569	< 0.001
Age	1.012	1.007–1.017	< 0.001	1.044	1.037–1.052	< 0.001
Male	1.321	1.115–1.567	0.001	0.331	0.261–0.419	< 0.001
BMRm	1.002	1.001–1.002	< 0.001	1.003	1.003–1.004	< 0.001
ALT	1.019	1.014–1.023	< 0.001			
AST	1.031	1.024–1.038	< 0.001	1.038	1.030–1.046	< 0.001
CRP	1.016	1.008–1.025	< 0.001			
WBC	1.085	1.045–1.126	< 0.001	1.096	1.049–1.145	< 0.001
Platelet	0.997	0.996–0.998	< 0.001	0.997	0.996–0.999	0.001
Diabetes (1,0)	0.382	0.307–0.475	< 0.001	0.444	0.347–0.567	< 0.001

*Note:* MASLD, metabolic dysfunction–associated steatotic liver disease; BMRh, basal metabolic rate calculated using the Harris–Benedict equation; BMRm, basal metabolic rate calculated using the Mifflin–St Jeor equation; ALT, alanine aminotransferase; AST, aspartate transaminase.

Abbreviations: CI, confidence interval; CRP, C‐reaction protein; WBC, white blood cell.

Two new models were developed based on the multivariate analysis: Model_BMRh(y = 1) = (exp  (−8.954 + 0.042∗age − 1.247∗male(0, 1) + 0.003∗BMRh + 0.037∗AST + 0.088∗WBC − 0.003∗PLT − 0.810∗diabetes(0, 1)))/(1 + exp  (−8.954 + 0.042∗age − 1.247∗male(0, 1) + 0.003∗BMRh + 0.037∗AST + 0.088∗WBC − 0.003∗PLT − 0.810∗diabetes(0, 1))) and Model_BMRh(y = 1) = (exp  (−9.332 + 0.043∗age − 1.106∗male(0, 1) + 0.003∗BMRh + 0.037∗AST + 0.092∗WBC − 0.003∗PLT − 0.813∗diabetes(0, 1)))/(1 + exp  (−9.332 + 0.043∗age − 1.106∗male(0, 1) + 0.003∗BMRh + 0.037∗AST + 0.092∗WBC − 0.003∗PLT − 0.813∗diabetes(0, 1))). The AUROC of Model_BMRh and Model_BMRm were 0.772 and 0.771, which was significantly higher than those of APRI, FIB‐4, and GPR (AUROC = 0.597, 0.585, and 0.638, respectively; all *p* < 0.001) (Figure 1(b)). With an optimal cutoff value of 0.137, Model_BMRh achieved 73.08% sensitivity and 68.91% specificity (Youden index = 0.420). Similarly, with an optimal cutoff value of 0.153, Model_BMRm achieved 69.64% sensitivity and 73.23% specificity (Youden index = 0.429).

### 3.4. Development of Two New Models for Predicting Significant Fibrosis in NAFLD

Univariate analysis identified age, BMRh, ALT, AST, CRP, WBC, platelet count, and diabetes as factors associated with significant fibrosis (all *p* < 0.01). Multivariate analysis confirmed that age, BMRh, AST, WBC, and diabetes (all *p* < 0.05) were independent risk factors for significant fibrosis. When BMRm was substituted for BMRh, age, BMRh, AST, WBC, and diabetes remained independent predictors of significant fibrosis (all *p* < 0.05) (Table 4).

**TABLE 4:** Risk factors for significant fibrosis in participants with NAFLD.

Variables	Univariate			Multivariate		
	Odds ratio	95% CI	*p*	Odds ratio	95% CI	*p*
Age	1.013	1.004–1.022	0.003	1.036	1.024–1.048	< 0.001
Male	0.874	0.658–1.161	0.353			
BMRh	1.002	1.001–1.002	< 0.001	1.002	1.002–1.003	< 0.001
ALT	1.024	1.016–1.032	< 0.001			
AST	1.041	1.027–1.055	< 0.001	1.038	1.023–1.053	< 0.001
CRP	1.021	1.007–1.035	0.003			
WBC	1.136	1.066–1.210	< 0.001	1.095	1.020–1.176	0.012
Platelet	0.997	0.995–0.999	0.008			
Diabetes (1,0)	0.288	0.213–0.319	< 0.001	0.342	0.242–0.483	< 0.001
Age	1.013	1.004–1.022	0.003	1.038	1.026–1.051	< 0.001
Male	0.874	0.658–1.161	0.353			
BMRm	1.002	1.002–1.002	< 0.001	1.003	1.002–1.003	< 0.001
ALT	1.024	1.016–1.032	< 0.001			
AST	1.041	1.027–1.055	< 0.001	1.039	1.024–1.054	< 0.001
CRP	1.021	1.007–1.035	0.003			
WBC	1.136	1.066–1.210	< 0.001	1.096	1.021–1.177	0.011
Platelet	0.997	0.995–0.999	0.008			
Diabetes (1,0)	0.288	0.213–0.319	< 0.001	0.345	0.244–0.486	< 0.001

*Note:* BMRh, basal metabolic rate calculated using the Harris–Benedict equation; BMRm, basal metabolic rate calculated using the Mifflin–St Jeor equation; ALT, alanine aminotransferase; AST, aspartate transaminase.

Abbreviations: CI, confidence interval; CRP, C‐reaction protein; NAFLD, nonalcoholic fatty liver disease; WBC, white blood cell.

Two new models were developed based on the multivariate analysis: Model_BMRh_NAFLD (*y* = 1) (exp  (−8.578 + 0.035∗age + 0.002∗BMRh + 0.037∗AST + 0.090∗WBC − 1.068∗diabetes(0, 1)))/(1 + exp  (−8.578 + 0.035∗age + 0.002∗BMRh + 0.037∗AST + 0.090∗WBC − 1.068∗diabetes(0, 1))) and Model_BMRm_NAFLD(*y* = 1) (exp  (−9.282 + 0.038∗age + 0.003∗BMRm + 0.038∗AST + 0.091∗WBC − 1.060∗diabetes(0, 1)))/(1 + exp  (−9.282 + 0.038∗age + 0.003∗BMRm + 0.038∗AST + 0.091∗WBC − 1.060∗diabetes(0, 1))). The AUROC of Model_BMRh_NAFLD and Model_BMRm_NAFLD were 0.780 and 0.782, which were significantly higher than those of APRI, FIB‐4, and GPR (AUROC = 0.611, 0.580, and 0.655, respectively; all *p* < 0.001) (Figure 2(b)). With an optimal cutoff value of 0.116, Model_BMRh_NAFLD achieved 76.23% sensitivity and 65.95% specificity (Youden index = 0.422). Similarly, with an optimal cutoff value of 0.129, Model (BMRm_AST_diabetes) achieved 74.89% sensitivity and 69.35% specificity (Youden index = 0.442).

### 3.5. Application of the New Models in Participants With Obesity, Overweight, and Normal Weight

For participants with MASLD and obesity, both the Model_BMRh and Model_BMRm had higher AUROCs (0.751 and 0.749) than APRI, FIB‐4, and GPR in predicting significant fibrosis (AUROC = 0.605, 0.607, and 0.623, respectively; all *p* < 0.001) (Figure 3(a)). However, among MASLD patients with overweight or normal weight, Model_BMRh, Model_BMRm, APRI, FIB‐4 and GPR demonstrated comparable diagnostic utility (all *p* > 0.05) (Figures 3(b) and 3(c)).

FIGURE 3Application of Model_BMRh and Model_BMRm for evaluating fibrosis in individuals with MASLD and obesity (a), overweight (b), and normal weight (c). BMRh, basal metabolic rate calculated using the Harris–Benedict equation; BMRm, basal metabolic rate calculated using the Mifflin–St Jeor equation; MASLD, metabolic dysfunction–associated steatotic liver disease; APRI, AST‐to‐PLT ratio index; FIB‐4, fibrosis‐4; GPR, gamma‐glutamyl transpeptidase to platelet ratio.

**Figure:**
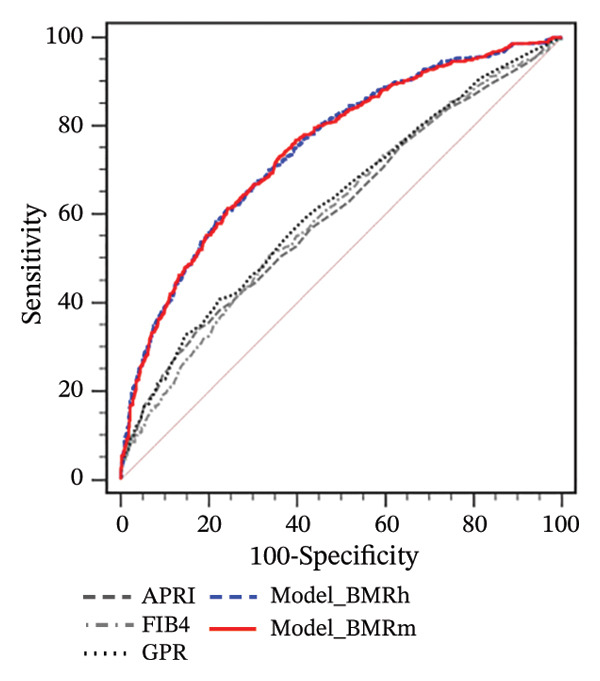
(a)

**Figure:**
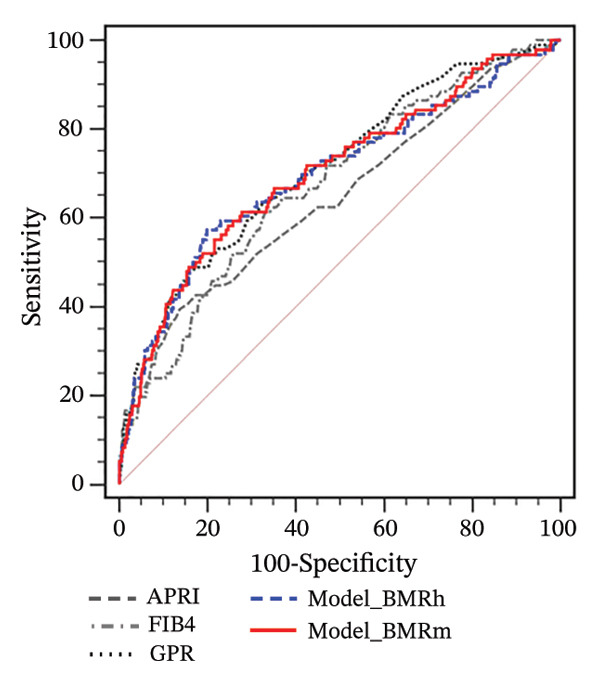
(b)

**Figure:**
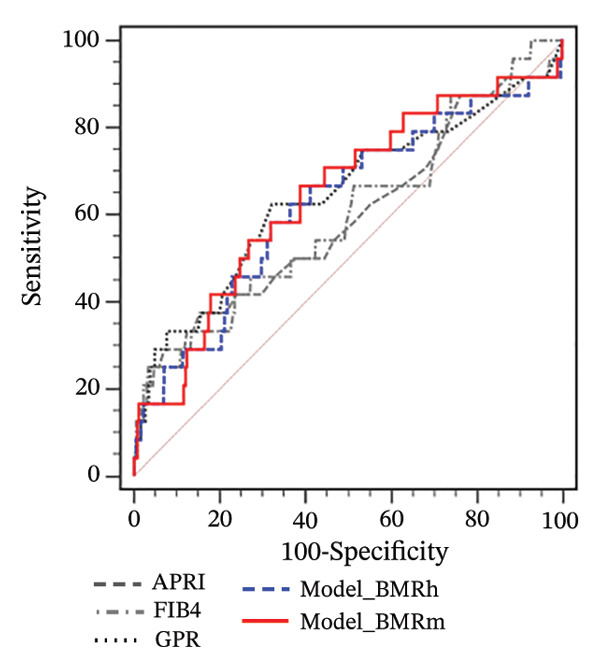
(c)

Consistent with the findings in MASLD, among participants with NAFLD and obesity, the AUROCs of Model_BMRh_NAFLD and Model_BMRm_NAFLD for predicting significant fibrosis were 0.749 and 0.754, respectively, which were significantly higher than those of APRI, FIB‐4, and GPR (AUROC = 0.622, 0.605, and 0.636, respectively; all *p* < 0.001) (Figure 4(a)). However, in individuals with NAFLD and overweight or normal weight, Model_BMRh_NAFLD and Model_BMRm_NAFLD showed no significant differences from APRI, FIB‐4, or GPR (all *p* > 0.05) (Figures 4(b) and 4(c)).

FIGURE 4Application of Model_BMRh_NAFLD and Model_BMRm_NAFLD for evaluating fibrosis in individuals with NAFLD and obesity (a), overweight (b), and normal weight (c). AST, aspartate transaminase; BMRh, basal metabolic rate calculated using the Harris–Benedict equation; BMRm, basal metabolic rate calculated using the Mifflin–St Jeor equation; NAFLD, nonalcoholic fatty liver disease; APRI, AST‐to‐PLT ratio index; FIB‐4, fibrosis‐4; GPR, gamma‐glutamyl transpeptidase to platelet ratio.

**Figure:**
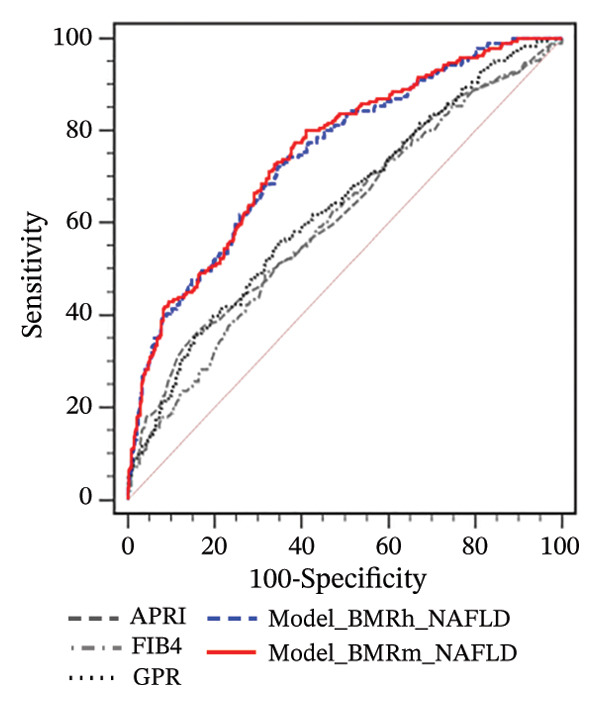
(a)

**Figure:**
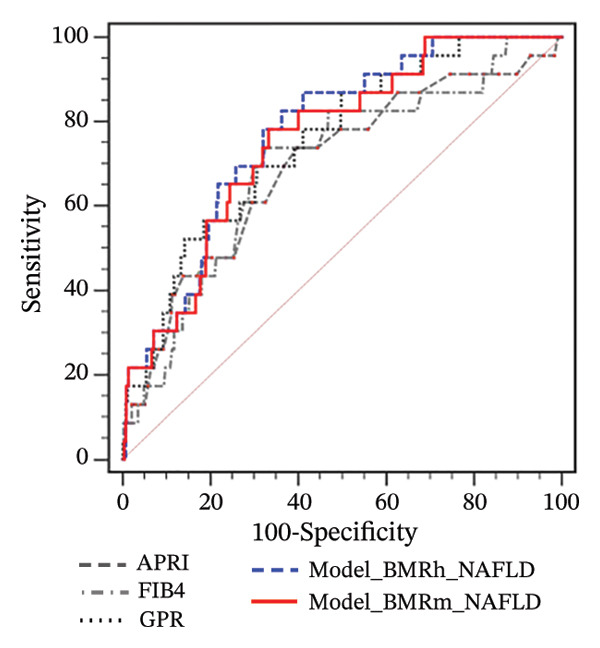
(b)

**Figure:**
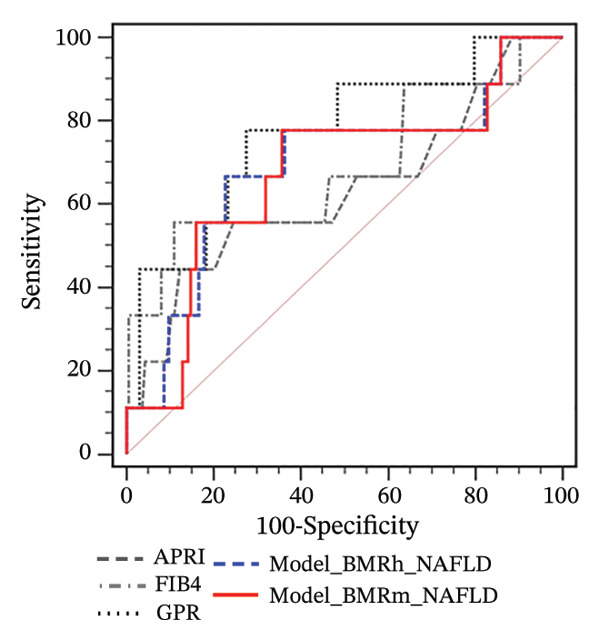
(c)

## 4. Discussion

This study investigated the predictive value of BMR for significant fibrosis. Analysis of data from 4184 individuals with MASLD and 1577 individuals with NAFLD revealed that both BMRh and BMRm were elevated in those with significant fibrosis and were independent risk factors. The models incorporating either BMRh or BMRm showed higher predictive accuracy than APRI, FIB‐4, and GPR for identifying significant fibrosis in obesity individuals with NAFLD or MASLD.

Growing evidence has demonstrated a strong association between diabetes and NAFLD or MASLD. In Asian Indian individuals with T2DM, approximately one‐third were found to have significant fibrosis [20]. In this study, 14.1% (223/1577) of individuals with FibroScan‐confirmed NAFLD had significant fibrosis. Notably, diabetes was an independent risk factor for fibrosis progression, emphasizing the importance of fibrosis assessment in individuals with NAFLD or MASLD, particularly those with diabetes.

Due to the invasive nature of liver biopsy, its use for monitoring fibrosis progression is limited. Currently, transient elastography using FibroScan provides an effective alternative for fibrosis evaluation. This study showed that BMRh and BMRm were higher in individuals with significant fibrosis. Compared with commonly used NSSs such as APRI, FIB‐4, and GPR, the models incorporating BMR provided stronger predictive performance. These findings support the use of BMR‐based models as a practical and accessible approach for identifying significant fibrosis in clinical settings.

Although BMR appears to play a role in fibrosis progression, the underlying mechanisms remain unclear. NHANES data showed a positive correlation between BMR and LSM, and BMR was higher in those with significant fibrosis. Since fat‐free mass alone may not fully explain this association, further investigation is needed. Previous studies have linked subclinical hypothyroidism and low thyroid function with advanced fibrosis and MASLD [21–25]. Reduced intracellular thyroid hormone levels and diminished THR activation have been observed in MASLD, and selective thyromimetics targeting THR‐β have demonstrated promising liver‐specific activity in clinical trials [26]. In addition, BMR has been found to correlate negatively with FT3 and TSH in patients with NAFLD and T2DM [27]. These findings suggest potential interactions between BMR and thyroid hormones that may influence energy and lipid metabolism. Future studies exploring BMR dynamics during THR‐β agonist therapy may offer new insights. Furthermore, BMR‐modifying strategies such as high‐intensity interval training, resistance training, increased lean muscle mass, sleep regulation, and stress management may enhance the therapeutic effect of THR‐β agonists.

In participants with MASLD—regardless of obesity, overweight, or normal weight—both BMRh and BMRm were positively correlated with BMI and negatively correlated with age. Among individuals with MASLD and obesity, both BMRh and BMRm showed positive correlations with LSM and CAP. These findings suggest that BMR may be primarily influenced by age and BMI, tending to be lower in older adults and higher in individuals with obesity. In the context of obesity, variations in BMR may influence intrahepatic fat distribution and the severity of liver fibrosis. In contrast, among overweight participants with MASLD, neither BMRh nor BMRm demonstrated a significant correlation with LSM or CAP. In normal‐weight participants with MASLD, both BMRh and BMRm were positively correlated with LSM but showed no significant association with CAP. These results imply that LSM may also be modulated by additional factors, such as systemic inflammation, alterations in gut microbiota, metabolic dysfunction, and insulin resistance. The complex interplay among gut microbiome–induced nonspecific inflammation, metabolic disorders, and insulin resistance is hypothesized to modulate the relationship between BMR and liver fibrosis, an area that merits further study. Together, these insights suggest that intervention strategies to mitigate the progression of MASLD‐related liver disease should be tailored to different population subgroups.

Given that MASLD is a multifactorial disease, potential confounders—such as dietary patterns, physical activity, weight management, and subclinical thyroid disease—may influence both BMR and the progression of fibrosis. The relationship between BMR and LSM differs among individuals with obesity, overweight, and normal weight. Given that BMR is largely determined by age and BMI, other factors—such as systemic inflammation or metabolic dysfunction—may play a more critical role in LSM progression in individuals with lean MASLD. Moreover, dynamic changes in BMI or BMR, rather than their baseline levels, may also contribute to the progression of liver fibrosis. To the best of our knowledge, aside from FibroScan, noninvasive methods for assessing the degree of liver fibrosis in lean MASLD remain limited. These findings highlight the need for further research into the mechanisms of hepatic fibrogenesis and the development of evaluation methods specifically tailored to this population.

This study has several limitations. First, the cross‐sectional design prevents evaluation of long‐term changes in BMR and liver fibrosis. Second, BMR was estimated using formula‐based methods, which may introduce calculation bias. Although indirect calorimetry is the standard for BMR measurement and body composition is commonly assessed by tetrapolar BIA, these tools are not widely available in community healthcare settings. Thus, the convenient and noninvasive methods used in this study may offer greater clinical applicability. Third, given the known relationship between thyroid hormones and NAFLD or MASLD progression, further research is warranted to explore the interaction between BMR and hypothyroidism in individuals with significant fibrosis. Fourth, although FibroScan serves as an alternative to liver biopsy, it has limitations in clinical practice. Its diagnostic accuracy can be compromised by factors such as severe obesity, cholestasis, or elevated total bilirubin. Future studies employing biopsy‐proven fibrosis evaluation are warranted to validate and strengthen these findings.

## 5. Conclusions

BMR is elevated in individuals with MASLD or NAFLD and significant fibrosis. The model combining age, male gender, BMR, AST, WBC, platelet counts, and diabetes outperformed conventional NSSs in predicting significant fibrosis in individuals with MASLD and obesity.

**Nomenclature**
ALT: Alanine aminotransferase
AST: Aspartate transaminase
BMI: Body mass index
CAP: Controlled attenuated parameter
CRP: C‐reactive protein
BMRh: Basal metabolic rate calculated using the Harris–Benedict equation
BMRm: Basal metabolic rate calculated using the Mifflin–St Jeor equation
GGT: γ‐glutamyl transferase
NAFLD: Nonalcoholic fatty liver disease
MASLD: Metabolic dysfunction–associated steatotic liver disease
PLT: Platelet
WBC: White blood cell
NSSs: Noninvasive scoring systems
APRI: Aminotransferase‐to‐platelet ratio index
FIB‐4: Fibrosis‐4
GPR: Gamma‐glutamyl transpeptidase to platelet ratio
WC: Waist circumference
HbA1c: Hemoglobin A1c.

## Author Contributions

Yuan Xue and Xue‐ming Zhang were responsible for study design. Data were collected by Jing‐tao Qin, Xue‐ming Zhang, Yi‐shan He, Shu‐qin Zheng, and Yuan Xue. Data analysis was performed by Shu‐qin Zheng, Jing‐tao Qin, Xue‐ming Zhang, and Yuan Xue. The manuscript was written by Shu‐qin Zheng, Jing‐tao Qin, Xue‐ming Zhang and Yuan Xue. Shu‐qin Zheng and Jing‐tao Qin contributed equally to this study.

## Funding

This study was supported by the Research Foundation of Jiangsu Province Administration of Traditional Chinese Medicine (Grant no. MS2023088) and the Leading Talent of Changzhou “The 14th Five‐Year Plan” High‐Level Health Talents Training Project (2022CZLJ021).

## Disclosure

All authors have read and approved the final manuscript.

## Ethics Statement

The survey protocol was approved by the National Center for Health Statistics Ethics Review Committee, and the database is publicly available. All participants provided written informed consent.

## Consent

Please see the Ethic Statement.

## Conflicts of Interest

The authors declare no conflicts of interest.

## Data Availability

The datasets supporting the conclusions of this article are available at https://www.cdc.gov/nchs/nhanes.
